# CD39 as a marker of pathogenic CD8+ T cells in cancer and other chronic inflammatory diseases

**DOI:** 10.18632/oncoscience.404

**Published:** 2018-04-29

**Authors:** Fernando Pablo Canale, María Cecilia Ramello, Carolina Lucía Montes

**Affiliations:** Centro de Investigaciones en Bioquímica Clínica e Inmunología (CIBICI-CONICET), Departamento de Bioquímica Clínica, Facultad de Ciencias Químicas, Universidad Nacional de Córdoba, Haya de la Torre y Medina Allende, Ciudad Universitaria, Córdoba 5000, Argentina

**Keywords:** CD39, Exhaustion, CD8+ T cells, Cancer, Chronic Inflammation

CD8+ T cells have long been considered crucial in the immune response against tumors. However, in the last decade a subpopulation of exhausted CD8+ T cells has been described which exhibit a dysfunctional phenotype and contribute to poor immune control of tumors and chronic viral infections. These findings led to the design of immunotherapies consisting in antibodies that block inhibitory receptors expressed by exhausted T cells in order to reinvigorate their effector function (known as “immune checkpoint blockade therapies”). This approach caused a great impact in clinics since an unprecedented number of treated cancer patients were able to recover the anti-tumor response mediated by CD8+ T cells [1]. Nevertheless, the durability of this enhancement is currently unknown, which raises the need to find new biomarkers to monitor the immune response against tumors. Moreover, these results also motivate the search for new immune checkpoints expressed by CD8+ T cells to design new immunotherapeutic approaches.

Many studies have highlighted the role of the ecto-enzyme CD39 in regulating immunity during chronic inflammatory diseases. This surface-expressed molecule is involved in the conversion of pro- inflammatory extracellular ATP to adenosine, a very potent immunosuppressive factor. CD39 can be expressed by a variety of cells, but in the tumor microenvironment it has been mostly attributed to Foxp3+ regulatory T cells [2]. However, a few years ago Parodi et al. described the presence of CD8+ T cells expressing CD39 in tumor biopsies of patients with different types of cancer [3].

In the recently published article “CD39 expression defines cell exhaustion in tumor-infiltrating CD8+ T cells” in Cancer Research we showed that CD39 is expressed by a subset of CD8+ T cells which exhibit an impairment in effector cytokines production and high expression of inhibitory receptors, all features of exhausted T cells [4]. These CD39+CD8+ T cells were present in all three murine cancer experimental models used (melanoma, mammary carcinoma and fibrosarcoma) and in primary tumors and metastatic lymph nodes from breast cancer patients. In contrast, they were absent or poorly represented in non-metastatic lymphoid organs and peripheral blood. Notably, the frequency of a subset of tumor-infiltrating CD8+ T cells with high expression of the ecto-enzyme (CD39high) increased with tumor growth in mice, indicating that the continued exposure to the tumor microenvironment would be responsible for the generation of these cells. The findings highlight the value of CD39 as a maker for exhausted CD8+ T cells in cancer. These results also propose that exhausted T cells are not merely inert cells which have lost their effector functions since they could also acquire a regulatory phenotype that favors tumor progression. A high capacity to hydrolyze extracellular ATP by tumor-infiltrating exhausted CD8+ T cells further supports this hypothesis.

**Figure 1:**
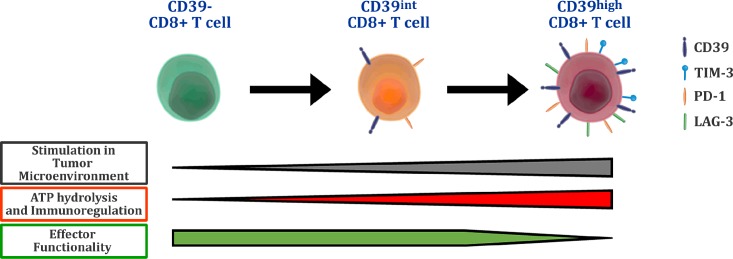
The tumor microenvironment elicits a subset of functionally exhausted CD8+ T cells that express the modulatory ectoenzyme CD39

In addition, we observed that more immunogenic tumors induce a higher frequency of CD39highCD8+ T cells. This was observed by comparing tumor-infiltrating CD8+ T cells from mice bearing B16F10 and B16F10- OVA tumors. The latter promote a more robust CD8+ T cell response, but at the same time a higher induction of the ecto-enzyme. Similar observations have been made in human cancers where highly immunogenic tumors promote the expression of inhibitory receptors and accumulation of exhausted T cells. It is in this type of tumors where checkpoint blockade therapies show better results (Figure 1).

All the conclusions from this work are consistent with observations made by other groups who described the presence of CD39+CD8+ T cells in different pathological scenarios. Gupta et al. observed in peripheral blood from patients with chronic viral infections that CD8+ T cells that are specific for HIV or HCV antigens showed higher expression of CD39 than CD8+ T cells specific for other viruses that cause acute infections [5]. They also observed that these cells showed high expression of PD-1 and exhibited a transcriptional signature consistent with exhaustion. A posterior work by other group showed that an elevated frequency of CD39+CD8+ T cells in patients co-infected with HIV and *M. tuberculosis* correlated with early mortality, despite anti-retroviral treatment initiation [6]. In another context, Bai et al. found an increase in the frequency of CD39+CD8+ T cells in peripheral blood and lamina propria from patients with Crohn disease, compared to healthy donors. They hypothesized that these cells could be harmful for patients due to their sustained production of IFNγ, a cytokine involved in the chronic inflammation of the disease [7]. Collectively, all these studies show that CD39+CD8+ T cells may represent a pathogenic cell subset in chronic inflammatory diseases, which could be monitored for prognosis and patient´s follow up.

Noble et al. studied CD39+CD8+ T cells in a quite different scenario. They described the induction of these cells in mice that were administered with tolerogenic doses of ovalbumin [8], and proposed that they were involved in the modulation of the response against the foreign antigens. The work suggests a different angle of study of CD39+CD8+ T cells, in which their induction could be beneficial in the context of autoimmune diseases and allergies.

All these works together are in contrast with the anti-viral and anti-tumor functions that are usually attributed to CD8+ T cells. Particularly, our results point CD39 as a molecule that could be used to address the presence of exhausted T cells in cancer patients and follow their functionality during and after treatments. Even more, CD39 may emerge as a new immune checkpoint, which could be targeted to restore CD8+ T cell tumor immunity.
